# Monitoring the Heavy Metal Lead Inside Living Drosophila with a FRET-Based Biosensor

**DOI:** 10.3390/s20061712

**Published:** 2020-03-19

**Authors:** De-Ming Yang, Robeth Viktoria Manurung, Yu-Syuan Lin, Tai-Yu Chiu, Wei-Qun Lai, Yu-Fen Chang, Tsai-Feng Fu

**Affiliations:** 1 Microscopy Service Laboratory, Basic Research Division, Department of Medical Research, Taipei Veterans General Hospital, Taipei 11217, Taiwan; willie@gm.ym.edu.tw; 2 Institute of Biophotonics, School of Medical Technology & Engineering, National Yang-Ming University, Taipei 11221, Taiwan; f1288197@yahoo.com.tw (Y.-S.L.); taiyu.chiu@nhri.org.tw (T.-Y.C.); 3 Biophotonics and Molecular Imaging Research Center (BMIRC), National Yang-Ming University, Taipei 112, Taiwan; 4 Research Center for Electronics and Telecommunication, Indonesian Institute of Sciences (LIPI), Bandung 40135, Indonesia; robe007@lipi.go.id; 5 LumiSTAR Biotechnology, Inc., Taipei City 115, Taiwan; yu-fen.chang@lumistar.com.tw; 6 Department of Applied Chemistry, National Chi-Nan University, Nantou 54561, Taiwan; tffu@ncnu.edu.tw

**Keywords:** biosensor, heavy metal lead, Met-lead, FRET imaging, Drosophila

## Abstract

The harmful impact of the heavy metal lead on human health has been known for years. However, materials that contain lead remain in the environment. Measuring the blood lead level (BLL) is the only way to officially evaluate the degree of exposure to lead. The so-called “safe value” of the BLL seems to unreliably represent the secure threshold for children. In general, lead’s underlying toxicological mechanism remains unclear and needs to be elucidated. Therefore, we developed a novel genetically encoded fluorescence resonance energy transfer (FRET)-based lead biosensor, Met-lead, and applied it to transgenic Drosophila to perform further investigations. We combined Met-lead with the *UAS-GAL4* system to the sensor protein specifically expressed within certain regions of fly brains. Using a suitable imaging platform, including a fast epifluorescent or confocal laser-scanning/two-photon microscope with high resolution, we recorded the changes in lead content inside fly brains ex vivo and in vivo and at different life stages. The blood–brain barrier was found to play an important role in the protection of neurons in the brain against damage due to the heavy metal lead, either through food or microinjection into the abdomen. Met-lead has the potential to be a powerful tool for the sensing of lead within living organisms by employing either a fast epi-FRET microscope or high-resolution brain imaging.

## 1. Introduction

The seriousness of exposure to lead in the natural environment was originally identified from the relationship between the increase in the amount of lead detected in the air during the use of leaded gasoline and the decrease in the intelligence quotient (IQ) of children living near a gas station and highway roads [1,2,3,4,5]. Nowadays, leaded gasoline is banned in many countries; however, some countries still use it. Exposure to lead can have an impact on human health [3,5]. This impact can occur in several ways, including through leaded gasoline, incense smoke, hair dye, paints on the surface of toys or the walls of houses, Chinese herbal medicines, and water from old lead pipes that are awaiting replacement [2]. Lead ions have the capacity to mimic vital ions, such as calcium, zinc, and iron, to alter the normal functions of critical proteins. The affected proteins may be located on the cell membrane or within the cytosol of different tissues or systems, such as essential enzymes (protein kinase C and delta-aminolevulinic acid dehydratase) and channel/transporter proteins that transport these ions within cells [6]. In addition, the homeostasis of these ions cannot normally be maintained when lead exists inside or outside of the cells.

The blood lead level (BLL) represents the amount of lead circulating within the bloodstream, and its evaluation is the most popular clinical routine method for diagnosing lead poisoning before further intervention [4]. The accepted safe BLL value for adult humans is less than 10 μg/dL (500 nM). Below this value, no significant alterations in behavior can be found [4,5]. However, in children, problems with attention, executive function, visual–motor reasoning skills, vestibular proprioceptive control, and abnormal social behavior were found to be correlated to long-term exposure to a low amount of lead [4,5,7]. Currently, the BLL for children is 4 μg/dL (200 nM) or lower, even though chelation therapy is available. This fact has raised doubts over the accuracy of the safe BLL value, especially in young children. Indeed, the BLL alone does not provide sufficient information on the lead intoxication status of cells. Therefore, as the BLL continues to decline, laboratories may need to lower their analytical limits of detection (LODs) by improving the analytical process and making changes to technology. Improved methods are needed to precisely detect the actual amount of lead within the cells of our bodies and to alert us when we are in a dangerous environment.

### 1.1. Biosensors for Monitoring Lead in Living Organisms

For years, scientists have made and used tools to detect the contents of metal ions, such as lead, inside living cells during and after exposure to metals [8]. These tools may be commercially available fluorescent indicators, such as Indo-1, or laboratory-based and home-made probes, such as chemical and/or protein biosensors [9,10,11,12,13]. It is believed that, with the help of these tools, the mechanism by which lead damages the human body will one day be fully elucidated. We have put effort into achieving this aim by constructing the very first fluorescence resonance energy transfer (FRET)-based genetically encoded (GE) biosensor [14], Met-lead 1.59, for lead in 2012 [12]. The major sensing key of Met-lead, PbrR, is a member of the transcription regulator family MerR, and was originally found in *Cupriavidus metallidurans* [12,15]. This sensing key allows Met-lead 1.59 to have good metal ion selectivity, fair sensitivity (3 h of exposure to 10 μg/dL of lead, nearly 500 nM), and an acceptable dynamic range ((DR) a FRET ratio from 3.3 to 5.7, 1.72-fold) for sensing lead in live cells [12]. However, Met-lead 1.59 may not be appropriate for in vivo lead detection as it requires a high DR to obtain a significant increase in the ratio signal in situ [16]. The only way to solve this problem is to increase Met-lead 1.59’s DR (e.g., by more than 2-fold) and sensitivity (e.g., to lower than 50–100 nM) [17].

Optimizing GE biosensors is very challenging; time-consuming; and, most importantly, provides less repeatability according to previous experience [8,14,18,19,20]. GE biosensors for calcium, for example, required more than seven years of intense brainstorming and much effort to delicately modify with thousands of trial-and-error constructs, including the milestone circular permutation (cp) of green fluorescent protein (GFP) variants [18]. Finally, YC 3.6 was produced, which provides a sufficient increase in DR for practical application to calcium-sensing in live animals [19,20]. To date, mature, optimized GE biosensors for calcium have been successfully applied to several animal models, including fly, zebrafish, mouse, and rat models [21,22,23,24].

### 1.2. Systematic Model for Lead Biosensing

Recently, improved versions of Met-lead with a significant increase in DR have been developed in our laboratory [17]. This progress may provide us with a further opportunity to sense lead in vivo through transgenic models (e.g., Drosophila or Arabidopsis). In this study, we used a FRET ratio imaging platform to test the sensing ability of these improved versions of Met-lead inside certain tissues ex vivo and in vivo.

Many scientists have used Drosophila as an experimental animal model in developmental biology and other fields, such as neuroscience and toxicological science. Neurons are basic components of the brain that exert neuronal transmission activity. The similarity of human neurons and fly neurons allows flies to be a relatively easy-to-handle platform for the investigation of the neurotoxicity of lead [25,26]. In addition, the homeostasis of ions in extracellular fluids, the steady supply of nutrients and metabolites to the brain tissue, the solid barrier that protects neurons from various toxicants, and normal neuronal activity have all been found to be major contributions of the blood–brain barrier (BBB), which is critical to both human and fly brains [27,28]. However, some studies have failed to find any effects of lead on the behavior of flies under similar conditions to those that young children encounter, and therefore have raised doubts about whether flies are a suitable model for lead toxicology [26,29]. This may be due to the relatively short lifecycle of a fly from a larva to an adult (3 days) compared to that of a human from an infant to a young child and on to an adult (more than 15 years) under the protection of the BBB that deals with different levels of lead in the environment. The lack of suitable tools to monitor the changes in lead content inside the entire bodies of flies throughout their lifecycle as mentioned above is another possible reason. It is generally believed that, within adult human and fly brains, the BBB prevents the entry of lead into the brain and maintains normal physiological behavior.

## 2. Materials and Methods

### 2.1. Construction and Molecular Simulation of Met-Lead

The gene construct for Met-lead used in this study was adopted from a previously published paper [12] with some modifications [17]. Generally, the gene of the PbrR variant sensing key, which was originally found in *C. metallidurans* (CH34) [15] and used to specifically bind lead ions, was linked to that of the FRET backbone pair of YC 3.6, i.e., ECFP(ΔC11) and cp173Venus [12,17].

The molecular structure of the Met-lead protein containing PbrR-sensing peptides and the FRET protein pair (ECFP(ΔC11) and cp173Venus) was simulated using the PS2 protein structure prediction server (homology modeling) [30,31,32] and the Molecular Operating Environment (MOE, Chemical Computing Group Inc., with the help of Professor Wolfgang Fischer, Institute of Biophotonics, National Yang-Ming University) [33]. The input sequences of proteins of interest, together with the join chain, were exported to a .mol2 file and displayed by Pymol.

### 2.2. Sample Preparation

Cells from the HeLa human cancer cell line were obtained from the American Type Culture Collection (ATCC, Rockville, MD) and cultured according to the manual and a previous report [17]. Cells were seeded on 24 mm cover glasses (Deckglaser) coated with poly-L-lycine and transfected with a Met-lead gene as previously described [12,17]. The Met-lead-transfected cells were applied in lead biosensing using FRET ratio imaging after 2 days of transfection. In some experiments, 5 µM of ionomycin was used as artificial lead content inside and outside Met-lead-expressing samples [12,17,34].

Drosophila was used as a model system to examine Met-lead’s sensing ability. Fly stocks were raised on standard cornmeal food and housed at 25 °C with 70% relative humidity and a 12:12 hour light:dark cycle. GAL4 drivers were used to express Met-lead inside certain regions of the flies. *Elav-gal4* (#458) (purchased from the Bloomington Drosophila Stock Center, BDSC, University of Indiana, IN, USA) was used for pan-neurons, *Cha-gal4* was used for most of the cholinergic neurons, *R13F02-gal4* was used for neurons inside mushroom bodies [35], and *TH-gal4* was used for dopaminergic neurons [36]. The latter three lines were gifts from Professor Chia-Lin Wu (CGU, Taiwan). All brain images were acquired from flies using transgenic expression and the progeny obtained from crossing GAL4 with the *UAS-Met-lead* indicator line [17].

To perform in vivo FRET biosensing of lead inside adult fly brains, we put flies on a custom-made plate, removed the skull of each fly on that holder under an upright microscope, and then performed time-lapse ratio lead biosensing [17]. To image the central nervous system (CNS) of fly larvae, we cooled each intact larva on ice, plated it on a stereo or an upright FRET microscope, and then made quick image acquisitions.

For high-resolution imaging of fly brains (using a confocal or two-photon microscope), an ex vivo sample procedure was carried out [37]. Generally, adult or larva fly brains were carefully dissected and immersed in 4% paraformaldehyde pre-cooled on ice for 1 hour with gentle shaking. Subsequently, the brains were transferred to 2% PBST (phosphate-buffered saline (PBS) with 2% Triton X-100) for 30 min with gentle shaking and then placed in a vacuum chamber. The brain samples were mounted on coverslips and cleared with 15 μL of RapiClear (SunJin Lab, Co, Taiwan). Samples were stored in an electronic dry cabinet until imaging [17].

### 2.3. FRET Ratio Imaging

For fast FRET ratio imaging of lead within Drosophila fly brains, we used a stereo (Stemi 508, Zeiss) or an upright (BX-53, with a 4x or 20x objective lens, Olympus, Japan) microscope equipped with a 405 nm (commercially available for the stereo microscope) or 440 nm (an excitation filter for the upright microscope) light source and a W-View module (Gemini, Hamamatsu, Japan; with 542/27 nm filters for yellow fluorescent protein (YFP) and 483/32 nm filters for cyan fluorescent protein (CFP) and a complementary metal oxide semiconductor (CMOS) camera (ORCA-Flash4.0 LT, Hamamatsu, Japan) controlled by HCImage software. The fluorescent signals of cp173Venus and ECFP(ΔC11) from Met-lead-expressing samples, i.e., single cells or fly tissues (neurons inside the brain/CNS) ex vivo/in vivo, were quickly obtained from the FRET ratio image system [12,17].

For high-resolution, three-dimensional (3D) FRET ratio imaging of fly brains, we used a laser-scanning confocal microscope or a two-photon microscope. A 440 nm laser and an 850 nm two-photon laser were used as excitation sources within the confocal laser-scanning system (Zeiss LSM 880, with a 20x Numeric Aperture (NA) 0.8 objective lens, Germany) and the multiphoton microscope (Zeiss LSM 7 MP, with a 20X NA 1.0 water objective lens, Germany), respectively. Both YFP (530–630 nm) and CFP (460–500 nm) emission signals were acquired separately.

### 2.4. Data Analysis

ImageJ was employed to combine the ratios of fluorescent signals of cp173Venus (the YFP channel) and ECFP(ΔC11) (the CFP channel) through the “ratio plus” plug-in. Fluorescent signals were displayed with a rainbow color plate using the lookup table (LUT) to visualize the FRET biosensor (a color change from blue to red indicates the change from the lowest to the highest ratio value) within intact living cells or fly brains/CNSs. Other statistical data processing was performed as previously described [12,17].

## 3. Results

### 3.1. Structural Design of Met-Lead

**Figure 1:**
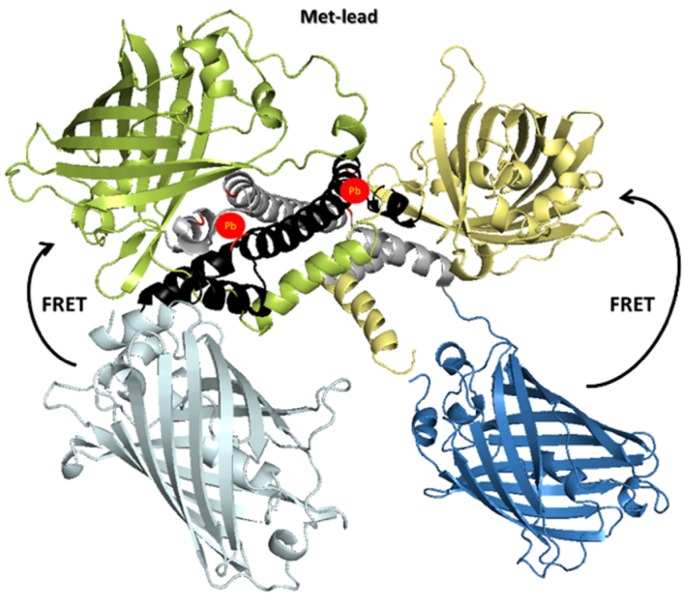
The predicted structure of Met-lead. Structural data from the MerR family member CueR [30] suggest that the lead sensing key (the PbrR protein, partial) is a dimer (shown in either gray or black for another strand in the homology modeling). Thus, the structure of Met-lead may also, in dimer form, bind two lead ions (red circles). The fluorescence resonance energy transfer (FRET) protein ECFP(ΔC11) is displayed in either blue or light blue, and cpVenus is displayed in yellow or green. The two lead-binding pockets at the opposite edges of each PbrR-sensing domain near the two FRET pairs are composed of the three cystine residues shown in red. Inside Met-lead, FRET events may happen upon the occurrence of lead ions through the driving of these two pairs together to favor energy transfer [12,17,19,20].

As a molecular structure presented from a 3D spatial perspective can provide additional information on the function of a sensor’s protein, we simulated the entire structure of Met-lead (Figure 1). According to the underlying mechanism of the conventional FRET approach, the general composition of Met-lead was designed to contain two parts: one lead-sensing motif (a Pb binding key; gray and black in Figure 1) and the pair of FRET proteins, i.e., ECFP(ΔC11) and cp173Venus (blue/light blue and yellow/green, respectively, in Figure 1). Similar to the classic FRET-based calcium biosensors cameleon and YC 3.6 [19,20], FRET events within Met-lead from ECFP(ΔC11) to cp173Venus can happen when lead ions exist. Specifically, around 440 nm of illumination can excite ECFP(ΔC11), and emission signals of both ECFP(ΔC11) and cp173Venus can subsequently be obtained using FRET ratio imaging.

### 3.2. Real-Time In-Cell Biosensing of Lead

**Figure 2:**
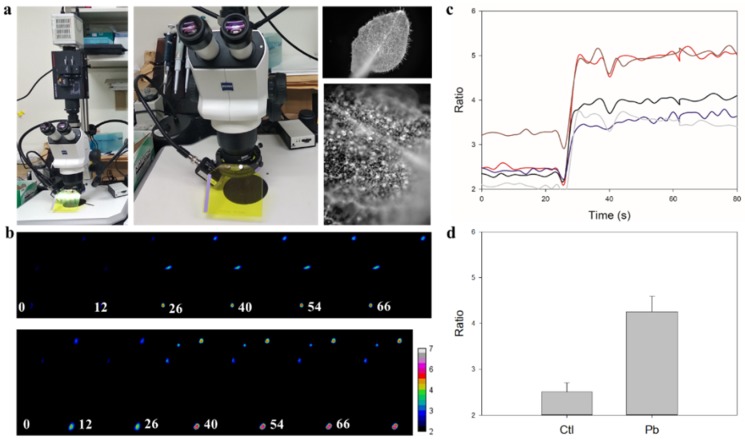
Biosensing of intracellular lead ions by Met-lead through a stereo microscope with a FRET module. (**a**) The established stereo-type FRET ratio imaging system for living samples expressing the Met-lead biosensor. Left: A view of the entire platform. Middle: The working distance for living samples. The excitation light is placed on the left side of the microscope’s main body to illuminate the sample. Right: A bright-field image of leaves showing the capability of the stereo microscope. The entire organism/organ/tissue (upper right) or single cells in situ (lower right) can be observed and imaged at different magnifications. (**b**) Two fields of view representing the time-lapse fluorescent ratio images shown in the rainbow color manner (cpVenus/ECFP(ΔC11); 6 graphs numbered with the time point in seconds in both the upper and lower panels) of single HeLa cells expressing Met-lead. During an 80 second recording, a buffer containing lead ions (10 μM) and ionomycin (5 μM) was added at around the 25 second time point. Images in the YFP channel and those in the CFP channel were combined through a ratio imaging process (see Materials and Methods). (**c**) The time-lapse emission ratio plots (lines in different colors) of five selected cells (image data extracted from panel (**b**)). (**d**) Bar graphs (the averaged ratio values for the five selected cells extracted from panel (**c**)) of the Ctl (control) within the first 0–20 s, compared with Pb within 40–80 s. In panel (**b**), the scale of the rainbow color ratio bar is 2–7.

Generally, an inverted FRET ratio imaging system is used for biosensing in single living cells [12,17,19,20]. To extend the application of Met-lead to different living organisms, such as fruit flies and plants, we for the first-time incorporated a FRET module into the stereo microscope (Figure 2a). Representative time-lapse emission ratio (cp173Venus/ECFP(ΔC11)) color images of Met-lead-expressing cells are shown in Figure 2b. Due to the presence of lead ions (10 μM, with the help of ionomycin, and at around 25 s after initiating the recording), the color of the ratio images of the five selected cells changed from blue to red (Figure 2b; Supplementary Movies 1 and 2). The emission ratio values within the same five selected cells all increased (Figure 2c). The bar graph shown in Figure 2d indicates that a significant increase in ratio occurred in response to lead. This example shows how Met-lead can be used to directly detect lead inside living cells using a stereo-type FRET ratio imaging system. Further details about the improved version of Met-lead, such as the sensitivity (LOD) and selectivity, can be found in a previous report [12] and elsewhere [17].

### 3.3. Fly Brains as Models for Lead Biosensing

**Figure 3:**
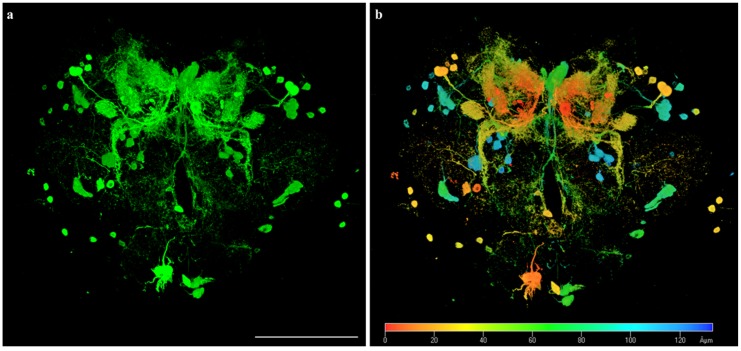
A three-dimensional (3D) optical image of a Drosophila fly brain (TH-gal4 > UAS-GFP) shown in a green fluorescent manner (**a**) and in depth color (**b**). In total, 161 optical sections were acquired and projected onto one plane. The scale bar is 100 μm. The depth color bar in panel (**b**) runs from 0 (red) to greater than 120 (blue) μm.

An important step in lead biosensing is the selection of a suitable model to demonstrate the biosensing ability of a biosensor. As Drosophila is a mature expression system (UAS-GAL4), we pretested 3D confocal images of brains through a combination of the GFP strain (UAS-GFP) and the dopaminergic neuron strain (TH-gal4) using the fine confocal laser-scanning microscope (Figure 3). Single neurons were observed on the cell centers of neurons, and their axons/dendrites are clearly shown in Figure 3a. Virtual sections of 3D reconstruction of fly brain images can display the spatial relationship of each neuron on the actual depth scale (Figure 3b, in at least 120 μm). The combination of high-resolution imaging (confocal laser scanning or two-photon imaging) and the Met-lead biosensor makes it possible to obtain a brand new blueprint of a fly’s brain circuitry that displays single lead-sensitive neurons as shown in Figure 3. This will further help us to understand more about the neurotoxic effects of lead on the brain using flies as an example animal model.

### 3.4. In Vivo Lead Biosensing within Adult Fly Brain Neurons

Next, we applied Met-lead to the transgenic Drosophila model system through the UAS-GAL4 strategy. Specific neurons within the mushroom body of a fly’s brain will express Met-lead with a combination of the fly strain R13F02-gal4 together with the Met-lead strain UAS-Met-lead (Supplementary Figure S1). Similar to R13F02-gal4 for the mushroom body of a fly’s brain, the Elav-gal4 and Cha-gal4 strains can be used for tissue-specific targeting of Met-lead in entire brain neurons and cholinergic neurons, respectively (Supplementary Figure S1).

**Figure 4:**
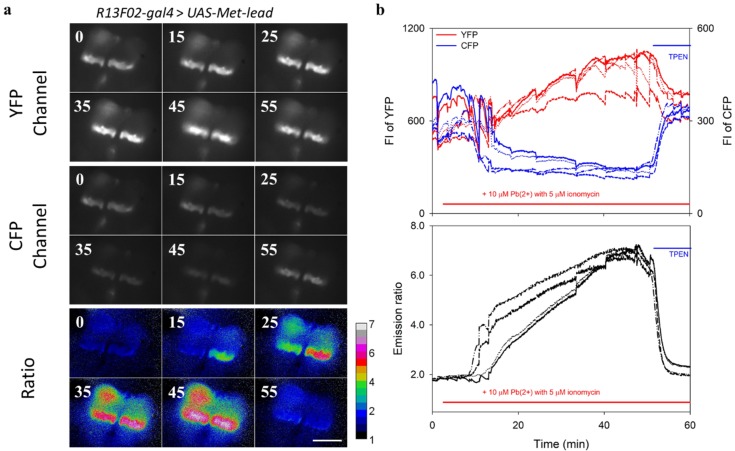
Application of Met-lead to adult Drosophila fly brains. (**a**) Images of Drosophila (R13F02-gal4 > UAS-Met-lead) fly brains were taken using an upright FRET platform. During a 60-minute recording, a buffer containing lead ions (10 μM) and ionomycin (5 μM) was added at around the 2 min time point (the red bar shown in the top and bottom graphs in panel (**b**)). The lead ion chelator TPEN (100 μM) was then added from around the 55 min time point until the end of the recording (the blue bar in panel (**b**)). Representative images of fly brains (the mushroom body) in the YFP channel (6 graphs numbered with the time points in minutes, top), in the CFP channel (middle), and in a ratio (YFP/CFP, bottom) are shown. The rainbow-color images were combined through a ratio imaging process. (**b**) Time-lapse plots from selected regions are displayed as fluorescent intensities (FIs) (YFP in red lines; CFP in blue lines, the top graph in panel (**b**)) and emission ratios (the bottom graph in panel (**b**)). The scale bar is 100 μm, and the rainbow color ratio bar is 1–7.

To demonstrate the in vivo lead-sensing ability of Met-lead within fly brains (R13F02-gal4 > UAS-Met-lead as an example), the skull of each fly’s head was removed so that the YFP/CFP fluorescent signals emitted from the neurons inside the mushroom body of each fly’s brain could be imaged (the YFP and CFP channels in Figure 4a). Ratio color images of Met-lead within the mushroom body of each fly’s brain are displayed in the lower panel of Figure 4a. In the 60-minute recording under ionomycin conditions, both the fluorescent intensities (FIs) of YFP/CFP (the upper panel in Figure 4b) and the ratio values (which increased from approximately 2 to close to 7; the bottom panel in Figure 4b) within selected regions of interest inside the fly brains clearly indicate the entry of lead ions into neurons. The membrane-permeable lead ion chelator N,N,N′,N′-tetrakis-(2-pyridylmethyl)ethylenediamine (TPEN, the blue bar in Figure 4b), which can simultaneously decrease the FIs of YFP (the red lines in Figure 2b) and increase those of CFP (the blue lines in Figure 2b), abolished the lead-induced increase in ratio value (back to 2), and thus directly proves such an increase in ratio representing a true lead-induced FRET event inside living fly brains. This TPEN-reversal is also visualized by the change in the color of the ratio images from red back to blue again (at 55 min in the bottom panel of Figure 4a). Similar experiments were carried out within another fly strain (Cha-gal4 > UAS-Met-lead) at a lower magnification (Supplementary Figure S2). The lead-induced increase in ratio resulted from the increase in the emission intensity in cp173Venus and the decrease in the emission intensity in ECFP(ΔC11).

### 3.5. Detecting Lead In Vivo within the CNS of Fly Larvae

**Figure 5:**
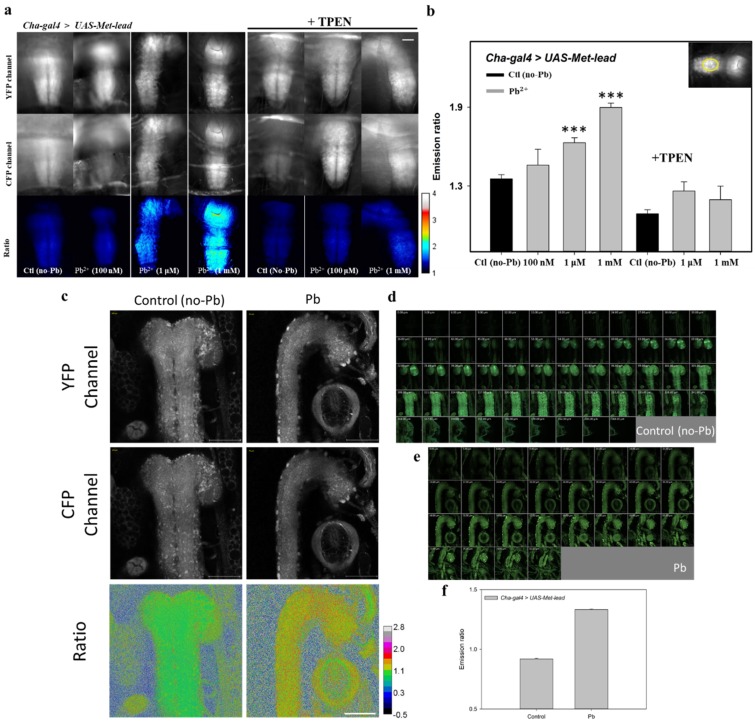
Application of Met-lead to the central nervous systems (CNSs) of Drosophila larvae using a fast epi-fluorescent (**a**,**b**) or two-photon (**c**–**f**) FRET imaging system. (**a**) Images of intact Drosophila larvae (Cha-gal4 > UAS-Met-lead) treated with different concentrations of lead (100 nM, 1 μM, and 1 mM) or TPEN in an ionomycin (5 μM) buffer. (**b**) The statistical ratio value of data from panel (**a**) is shown in the bar graph. A selected region of interest is shown in the inset. (**c**) Images of the CNS (cpVenus in the YFP channel; ECFP(ΔC11) in the CFP channel; cpVenus/ECFP(ΔC11) in a ratio) of Drosophila larvae (Cha-gal4 > UAS-Met-lead) were taken under a two-photon FRET microscope without (Control) or with lead (Pb, 10 μM). (**d**,**e**) A YC montage of merged images of the control (**d**) and lead (**e**) in different optical sections. The YC merged image’s resolution is further compared with ratio color images of each section in Supplementary Figure S3. (**f**) The statistical ratio value of the control and lead sets. The rainbow color ratio bar in panel (**a**) is 1–4. The rainbow color ratio bar in panel (**c**) runs from –0.5 to 2.8. The scale bar in panels (**a**) and (**c**) is 100 μm.

Within the CNSs of intact larvae (Cha-gal4 > UAS-Met-lead), Met-lead was shown to have the ability to monitor lead content using a fast FRET microscope as described above (Figure 4). Ionomycin-containing buffer with different concentrations of lead ions (100 nM, 1 μM, and 1 mM) was used to treat the intact larva, and some of the larvae were treated with TPEN to confirm that the increase in the emission ratio (from 1.3 to 1.9) was due to the existence of lead (Figure 5a,b).

In addition to the successful in vivo biosensing of lead in the CNSs of fly larvae, we used the two-photon microscope to obtain high-resolution 3D FRET ratio images of Met-lead within the CNSs of living fly larvae (Cha-gal4 > UAS-Met-lead, Figure 5c–e). Representative images of a larva’s CNS at the resolution of a single neuron in one optical section are shown in Figure 5c (the YFP and CFP channels are shown in the upper and middle parts of Figure 5c, respectively) and in Figure 5d,e in a montage series (a YC merged image of a larval CNS and an image of the Control in Figure 5d and an image of Pb in Figure 5e). The visualized ratio color images of the same representative section shown in the bottom panel of Figure 5c together with the statistical bar graph in Figure 5f reveal the significant change in ratio values (from 0.9 to 1.4) from control to lead (10 μM). The Supplementary Movies 3/5–6 and 4/7–8 show the ratio color/merged images of each optical section/3D stack of Control and Pb larval CNSs. The Supplementary Movies 9 and 10 show the 3D stack of larval CNSs ex vivo at the resolution of a single neuron and at different angles and magnifications. All of the larval CNS samples shown in Figure 5 were fixed before FRET ratio imaging was performed.

**Figure 6:**
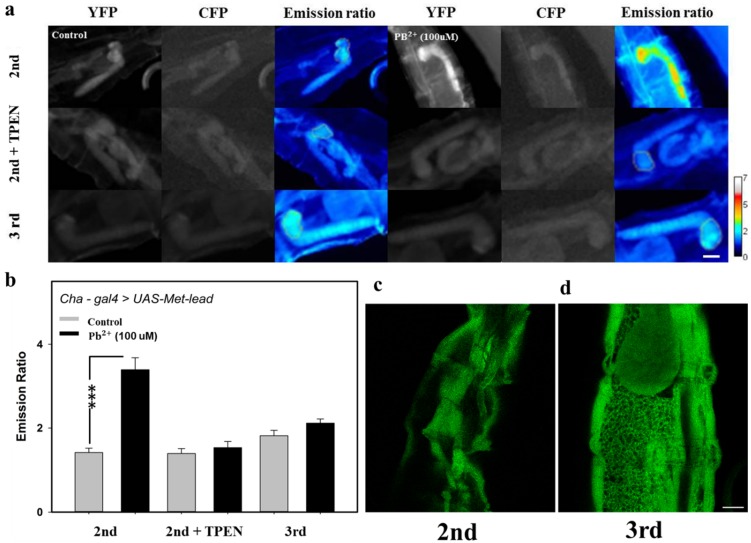
The role of the blood–brain barrier (BBB) in preventing the entry of lead into the brain/CNS of Drosophila larvae. (**a**) Images (cpVenus in the YFP channel; ECFP(ΔC11) in the CFP channel; cpVenus/ECFP(ΔC11) in an emission ratio) of the CNS of Drosophila larvae (Cha-gal4 > UAS-Met-lead) at different stages (2nd and 3rd) were taken using a fast epi-fluorescent FRET platform without (Control, microinjection of water into the abdomen) or with lead (Pb, 100 μM, microinjection into the abdomen). (**b**) Bar graphs of averaged emission ratios for the same treatments indicated in panel (**a**). Panels (**c**,**d**) are representative confocal images of the BBB (glial cells shown through the strain Repo-gal4 > UAS-GFP) at different stages (the 2nd stage in panel (**c**) and the 3rd stage in panel (**d**)). The rainbow color ratio bar in panel (**a**) is 0–7. The scale bar in panels (**a**), (**c**), and (**d**) is 100 μm.

Finally, we explored the role of the BBB within the larval CNS (Cha-gal4 > UAS-Met-lead) in protecting against lead invasion (Figure 6). Buffer containing lead (100 μM, without ionomycin) was micro-injected into the abdomen of a living fly larva at different stages (2nd and 3rd), and ratio imaging was performed after a 3-hour interval. The data shown in Figure 6 indicate that the CNS of the larva was invaded by lead only at the 2nd stage (the ratio color image in the upper-right part of Figure 6a compared to the control on the upper-left side and the 3rd stage on the lower side; a bar graph is shown in Figure 6b). The treatment with TPEN (the middle-right part in Figure 6a; the bar graph in Figure 6b) confirms that this increase in ratio value is due to lead ions that have traveled into the CNS region through circulation. Furthermore, the confocal images of the BBB, through the combination of the glia cell tissue-specific strain Repo-gal4 and the UAS-GFP strain, indicate that the maturation of the BBB was completed after the 3rd larval stage (Figure 6d, compared with the immature structure shown in Figure 6c). Taken together, the results shown in Figure 6 imply that the BBB plays an important role in preventing the entry of lead into the CNS region.

## 4. Discussion

To obtain knowledge about the effects of the heavy metal lead on living cells, we previously developed a novel FRET-based lead biosensor, Met-lead, for single living cells [12]. Here, using the optimized version of Met-lead [17], we detected lead inside the bodies of living animals, both at a young and adult age, through two types of FRET platforms, namely, stereo (Figure 2) and upright (Figure 4, Figure 5a, and Figure 6a) for fast acquisition, and two-photon imaging (Figure 5b) for high-resolution single-cell recordings. This study’s breakthrough is that the lead content inside different types of neurons within the brain/CNS of transgenic Drosophila expressing Met-lead was able to be successfully measured and displayed in vivo and ex vivo using suitable imaging setups.

Regarding the easy-to-use Drosophila expression system, the transgenic UAS strain (*UAS-Met-lead*) together with the GAL4 strains (*Elav-gal4*, *Cha-gal4*, *R13F02-gal4*, *TH-gal4*, and *Repo-gal4* for pan-neurons, cholinergic neurons, mushroom bodies, dopaminergic neurons, and glia cells/BBB, respectively), allows scientists to monitor the lead content inside specific tissues. This expression system may help us to trace the amount and traffic of lead at certain locations of living animals, and, hopefully, in the future, we can use it to explore possible lead-sensitive neurons within brain circuitry at the resolution of a single neuron [37].

To clarify the role of the BBB during exposure to lead in childhood, the lead biosensing data from fly larvae provided valuable information (Figure 5 and Figure 6). Similar to, but not completely the same as humans, the BBB of flies is formed by glial cells, of which the general marker is *Repo* (Figure 6c,d) [38,39]. There are seven types of glial cells in flies; the function of one of these types is nutrient transportation, and the function of another (subperineurialglial, SPG) is to seal the nervous system against hemolymphs through the formation of septate junctions. The maturation of the BBB in flies during neuronal development is completed around the middle of the 3rd larval stage under the regulation of the G-protein-coupled receptor (GPCR) signaling pathway and with contributions from the three critical proteins *Moody*, *Nrg*, and *Nrx* [40,41,42]. In this study, *Repo-gal4*, in combination with *UAS-GFP*, was used to reveal that the maturation of the BBB remains incomplete at the 2nd larval stage (Figure 6c) and will not be completed until the 3rd larval stage (Figure 6d).

The importance of the protective effect that BBB maturation in flies has against the invasion of the heavy metal lead into cholinergic neurons within the CNS of fly larvae was clearly observed through *Cha-gal4* in combination with *UAS-Met-lead* (Figure 6a,b). The integrity of an intact BBB is therefore believed to be very important to the prevention of contact with toxicants, such as exposure to lead ions in the external environment [4,25,29,40,41,42]. With the *UAS-Met-lead* strain (Figure 4, Figure 5 and Figure 6) [17], BBB mutant (defect) fly strains, such as *Moody*, *Loco*, and *Nrg*, can be combined with specific neuronal expression strains, such as *Elav-gal4*, *Cha-gal4*, *TH-gal4*, and *R13F02-gal4*, to reinvestigate the impacts of lead on fly brains in the future. This would allow us to track the lead ions inside fly brains, either at the larval or adult stage, using suitable FRET ratio imaging platforms as demonstrated in this study.

Our results suggest that the base ratio level of Met-lead in the different tested living organisms was consistent (between close to 2 and close to 3). Examples are single cells in the stereo microscope (Figure 2c, around 2.5) and single cells in the inverted microscope (around 2.5 to close to 3) [17]; adult/larva fly brain/CNS (in vivo) alive in the upright microscope (Figure 4b and Figure 6b, around 2.0 and 1.9); and leaves in the upright microscope (around 2.1) [17]. However, all of the base ratio values in the fixed samples were found to be around 1 to 1.3 (CNS of fly larvae in Figure 5b,f). Nevertheless, we were able to observe an increase in the Pb-induced FRET ratio and use TPEN to confirm that the increase in the ratio was due to Pb (Figure 5b). We suspect this problem to be due to the fixation procedure (in vivo or ex vivo). It could also influence the DRs. It is therefore necessary to successfully detect lead inside living samples using various FRET ratio platforms when samples are alive. However, living samples will still move, even when they are under anesthetic. We need to figure out how to avoid this problem, so that we can obtain super-fine single-cell images of live animals in vivo using a two-photon FRET imaging system. Our future goal is to overcome this challenge.

In conclusion, the results of this study demonstrate the practical application of Met-lead to a living system. The data here support a view that Met-lead is a workable tool for advanced methods for investigating lead. The general analytical methods for measuring the lead level in blood or other tested samples are graphite furnace atomic absorption spectrometry and inductively coupled plasma mass spectrometry. Both methods use complicated sample preparation procedures. The approach developed in this study not only provides information on the lead content inside living cells, but can also be applied to detect lead in the environment (an LOD of ~10 nM (~2 ppb)) [17].

## Supplementary Materials

The following are available online at https://www.mdpi.com/1424-8220/20/6/1712/s1. Figure S1: Expression pattern of GFP and Met-lead in adult Drosophila brains. Different types of neurons expressing GFP (upper part, *Elav-gal4 > UAS-GFP*; *R13F02-gal4 > UAS-GFP*; *Cha-gal4 > UAS-GFP*) or expressing Met-lead (lower part, *Elav-gal4 > Met-lead*; *R13F02-gal4 > UAS-Met-lead*; *Cha-gal4 > UAS-Met-lead*) were examined under a confocal microscope for 3D imaging. The scale bar is 50 μm. Figure S2: Practical application of Met-lead to adult *Drosophila* brains at a lower magnification (4x, compared with Figure 4 at 20x). (a) The in vivo biosensing of lead in *Drosophila* (*Cha-gal4 > UAS-Met-lead*) was carried out using an upright FRET imaging platform. During a 30-s recording, a buffer containing lead ions (10 μM) and ionomycin (5 μM) was added. Representative images of fly brains (cholinergic neurons) in the YFP channel (12 graphs numbered with the time points in minutes, upper), in the CFP channel (middle), and in a ratio (YFP/CFP, YC bottom) are shown. The rainbow color images were combined through a ratio imaging process. (b) and (c) The time-lapse plots from selected regions are displayed as fluorescent intensities (FIs) (YFP in red lines and CFP in blue lines (b) and emission ratios (c)). In (a), the scale bar is 50 μm and the rainbow color ratio bar is 1–3. Figure S3: YC montage images of Met-lead within the larval CNS of Drosophila. Control (upper) and lead-containing (lower) CNSs are shown in different optical sections in an RG merged manner or in a ratio color manner (the ratio color bar runs from –0.5 to 2.8). Supplementary Video S1: Biosensing of intracellular lead ions by Met-lead through a stereo microscope (Case 1). Supplementary Video S2: Biosensing of intracellular lead ions by Met-lead through a stereo microscope (Case2). Supplementary Video S3: Ratio color images of the control larval CNS in each optical section. Supplementary Video S4: Ratio color images of the Pb-treated larval CNS in each optical section. Supplementary Video S5: YC merged images of the control larval CNS in a 3D stack rotated on one axis. Supplementary Video S6: YC merged images of the control larval CNS in a 3D stack rotated on another axis. Supplementary Video S7: YC merged images of the Pb-treated larval CNS in a 3D stack rotated in one axis. Supplementary Video S8: YC merged images of the Pb-treated larval CNS in a 3D stack rotated on another axis. Supplementary Video S9: YC merged images of the control larval CNS ex vivo in a 3D stack rotated on one axis. Supplementary Video S10: YC merged images of the control larval CNS ex vivo in a 3D stack rotated on another axis.

## Abbreviations

BBB	blood brain barrier
BLL	blood lead level
CNS	central nervous system
CMOS	complementary metal–oxide–semiconductor
cp	circular permutation
CFP	cyan fluorescent protein
DR	dynamic range
FRET	fluorescence resonance energy transfer
FIs	fluorescent intensities
GE	genetically encoded
GFP	green fluorescent protein
GPCR	G protein-coupled receptor
IQ	intelligence quotient
LOD	limit of detection
LUT	look up table
NA	numeric aperture
PBS	phosphate-buffered saline
SPG	subperineurialglial
TPEN	N,N,N′,N′-tetrakis-(2-pyridylmethyl)ethylenediamine
YFP	yellow fluorescent protein
