# Supplementary material for: Monitoring the Heavy Metal Lead Inside Living Drosophila with a FRET-Based Biosensor
Source: Sensors (Basel). 2020 Mar 19;20(6):1712. doi: 10.3390/s20061712 (PMC7146181; doi:10.3390/s20061712)
Supplement: Supplementary file 1 [file sensors-20-01712-s001.pdf]

sensors

MDPI

Supporting Information

Monitoring the Heavy Metal Lead Inside Living Drosophila with a FRET-Based Biosensor

De-Ming Yang [1,2,3,#,*], Robeth Viktoria Manurung [4,#], Yu-Syuan Lin [2], Tai-Yu Chiu [2], Wei-Qun Lai [1,2], Yu-Fen Chang [5] and Tsai-Feng Fu [6]

1. Microscopy Service Laboratory, Basic Research Division, Department of Medical Research, Taipei Veterans General Hospital, Taipei 11217, Taiwan; willie@gm.ym.edu.tw
2. Institute of Biophotonics, School of Medical Technology & Engineering, National Yang-Ming University, Taipei 11221, Taiwan; f1288197@yahoo.com.tw (Y.-S.L.); taiyu.chiu@nhri.org.tw (T.-Y.C.)
3. Biophotonics and Molecular Imaging Research Center (BMIRC), National Yang-Ming University, Taipei 112, Taiwan
4. Research Center for Electronics and Telecommunication, Indonesian Institute of Sciences (LIPI), 40135 Bandung, Republic of Indonesia; robe007@lipi.go.id
5. LumiSTAR Biotechnology, Inc.,Taiwan; yu-fen.chang@lumistar.com.tw
6. Department of Applied Chemistry, National Chi-Nan University, Nantou 54561, Taiwan; tffu@ncnu.edu.tw

* Correspondence: dmyang@vghtpe.gov.tw

\# Co-first author to this work.

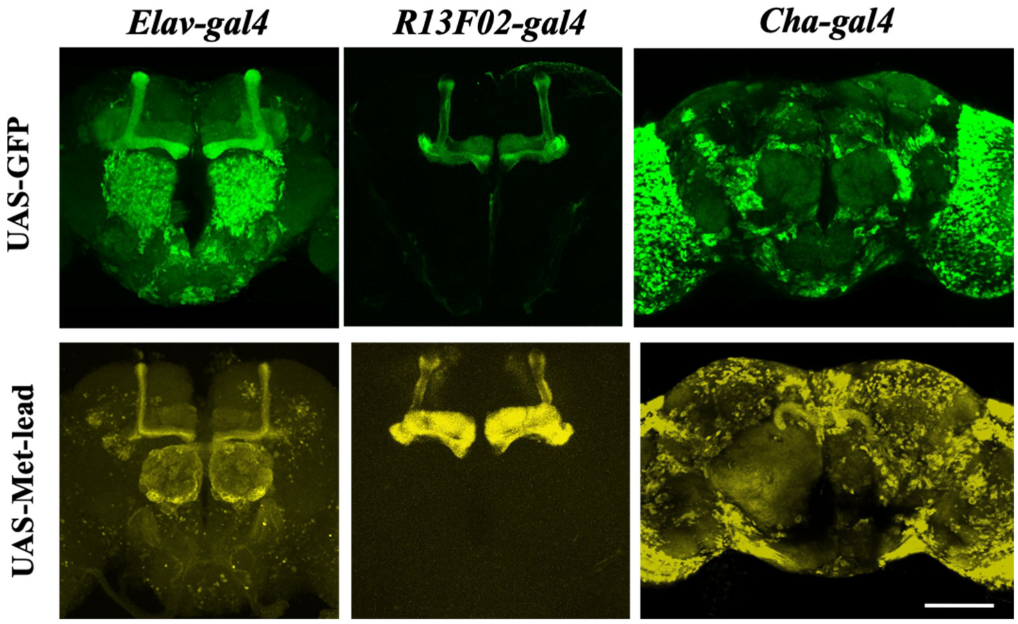

Supplementary Figure S1. Expression pattern of GFP and Met-lead on adult Drosophila brain. Different types of neurons expressing GFP (upper part, e.g., *Elav-gal4 > UAS-GFP*; *R13F02-gal4 > UAS-GFP*; and *Cha-gal4 > UAS-GFP*) or expressing Met-lead (lower part, e.g., *Elav-gal4 > Met-lead*; *R13F02-gal4 > UAS-Met-lead*; and *Cha-gal4 > UAS-Met-lead*) were took under confocal microscope for 3D imaging. The scale bar is 50 µm.

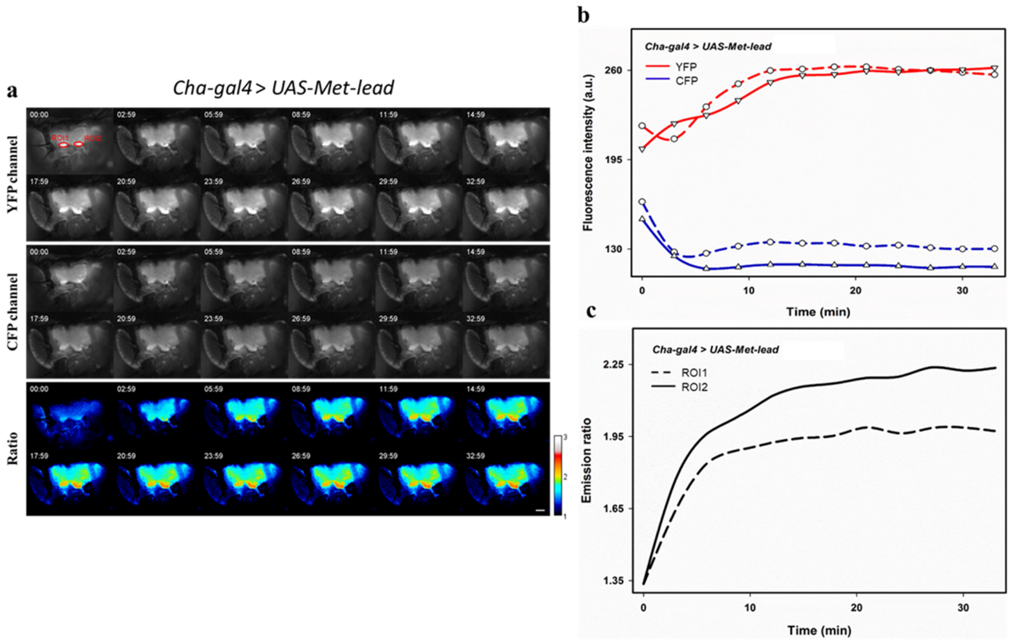

Supplementary Figure S2. Practical applications of Met-lead on adult Drosophila brain with lower magnification (4x, compared with Figure 4 in 20x). (a) The in vivo lead bio-sensing of Drosophila (*Cha-gal4 > UAS-Met-lead*) was carried out under an upright FRET image platform. During a 30 sec recording, a buffer containing lead ions (10 µM) and ionomycin (5 µM) was added. The representative fly brain (cholinergic neurons) images of YFP channel (12 graphs numbered with the time point in min, upper), CFP channel (middle), and ratio (YFP/CFP, bottom) are shown. The rainbow color images were transformed through a ratio image process. (b,c) The time-lapse plots from selected regions are displayed as fluorescent intensities, FIs (YFP in red lines; CFP in blue lines (b)) and emission ratios (c). In panel (a), the scale bar is 50 µm and the rainbow color ratio bar is from 1 to 3.

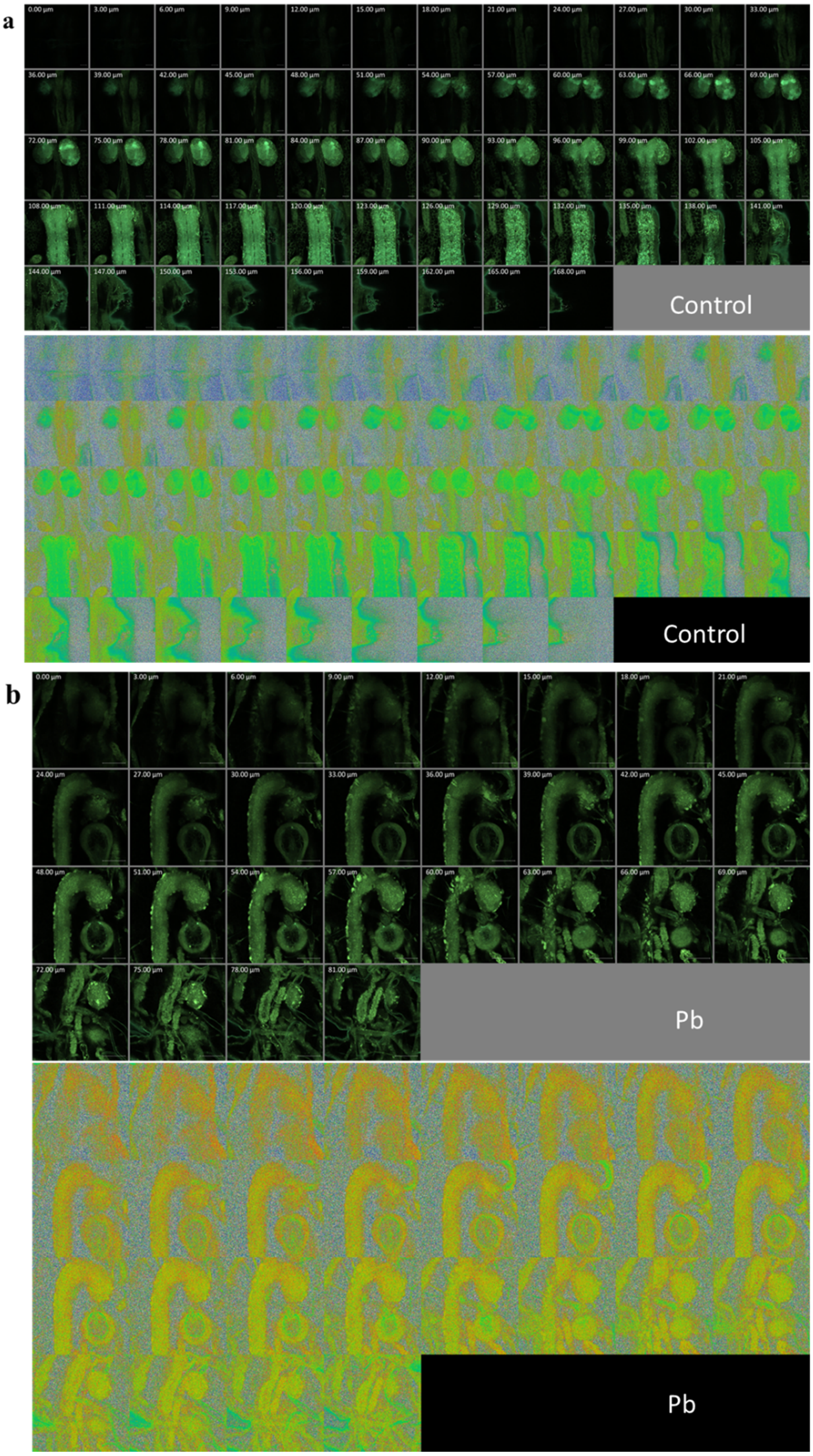

Supplementary Figure S3. The CY montage images of Met-lead within larva CNS of Drosophila. Control (upper) and lead (lower) CNS are shown in different optical sections in RG merged manner or in ratio color one (ratio color bar from −0.5 to 2.8).
